# *UBR5* Gene Mutation Is Associated with Familial Adult Myoclonic Epilepsy in a Japanese Family

**DOI:** 10.5402/2012/508308

**Published:** 2012-09-17

**Authors:** Takeo Kato, Gen Tamiya, Shingo Koyama, Tomohiro Nakamura, Satoshi Makino, Shigeki Arawaka, Toru Kawanami, Ikuo Tooyama

**Affiliations:** ^1^Department of Neurology, Hematology, Metabolism, Endocrinology and Diabetology, Faculty of Medicine, Yamagata University, 2-2-2 Iida-Nishi, Yamagata 990-9585, Japan; ^2^Genomic Information Analysis Unit, Advanced Molecular Epidemiology Research Institute, Faculty of Medicine, Yamagata University, 2-2-2 Iida-Nishi, Yamagata 990-9585, Japan; ^3^Molecular Neuroscience Research Center, Shiga University of Medical Science, Otsu 520-2192, Japan

## Abstract

The causal gene(s) for familial adult myoclonic epilepsy (FAME) remains undetermined. To identify it, an exome analysis was performed for the proband in a Japanese FAME family. Of the 383 missense/nonsense variants examined, only c.5720G>A mutation (p.Arg1907His) in the *UBR5* gene was found in all of the affected individuals in the family, but not in the nonaffected members. Such mutation was not found in any of the 85 healthy individuals in the same community nor in any of the 24 individuals of various ethnicities. The present study demonstrated an FAME-associated mutation in the *UBR5* gene, which is located close to the reported locus linked to Japanese FAME families.

## 1. Introduction 

Familial adult myoclonic epilepsy (FAME), or benign adult familial myoclonic epilepsy (BAFME), is a neurological disease of an autosomal-dominant inheritance, which is characterized clinically by adult onset of finger tremulous movement mixed with myoclonic jerks. Linkage studies have mapped FAME-associated loci to chromosome 8q23.3-q24.11 in Japanese families [1, 2], to chromosome 2p11.1-q12.2 in Italian [3–5] and Spanish [6] families, and chromosome 5p15.31-p15 in a French family [7]. Although the identification of the causal gene(s) for FAME is of great importance to the understanding of the molecular basis of the disease, gene(s) responsible for FAME has not been identified. Here, we report that a mutation of the gene encoding UBR5 (ubiquitin protein ligase E3 component *n*-recognin 5) is associated with FAME in a Japanese family.

## 2. Subjects and Methods 

### 2.1. Subjects

**Figure 1:**
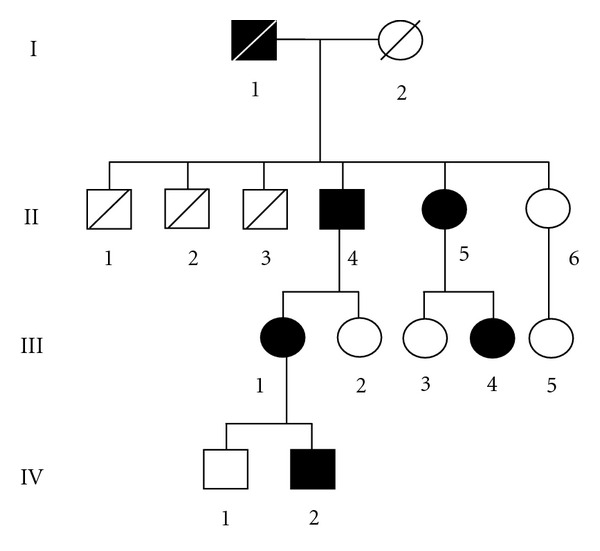
The pedigree of a Japanese family with FAME. The filled circles and rectangles indicate the affected women and men, respectively. Diagonal bar: deceased.

The pedigree of the present family with FAME is shown in Figure 1. All of the family members were born in a small town in Yamagata prefecture, about 400 km north of Tokyo, Japan. The family consisted of 6 individuals with FAME through four generations. Both men and women were affected with the disease, which is consistent with an autosomal-dominant inheritance. 

The proband (III-1 in Figure 1) was a 49-year-old woman who had noticed a shivering-like involuntary movement of the bilateral hands since the age of around 25 years. She had developed normally up to that time. The shivering-like movement consisted of arrhythmic, fine twitches at the hands, which was exaggerated by posture, feeling of strain, and fatigue. The involuntary movement was limited only in her hands, although it became slightly worsened with age, as compared with the condition at the onset. No additional neurological symptoms, including cerebellar ataxia, dementia, muscle weakness, sensory disturbance, or other involuntary movements, were observed. Her activity of daily living was completely independent. Brain MRI was also normal. The other affected members of the family showed virtually identical symptoms to the proband, although her cousin (44-year-old woman: III-4 in Figure 1) had an episode of generalized seizure at the age of 42 years, and her 74-year-old father (II-4) and 72-year-old aunt (II-5) showed a progression of myoclonic involuntary movements from the hands to all four extremities with aging, resulting in some difficulties in walking with assistance. 

All subjects gave written informed consent for genetic analysis. The study was approved by the Medical Ethics Committee of Yamagata University Faculty of Medicine.

### 2.2. Exosome Analysis on Next-Generation Sequencer (NGS)

For exosome analysis, genomic DNA from the proband was extracted and purified from whole blood using QIAamp DNA spin columns (QIAGEN N.V., The Netherlands). Exome capture was performed using the SureSelect Human All Exon System (Agilent Technologies, USA). The manufacturer's protocol for this system (Illumina Paired-End Sequencing Library Prep, version 1.0.1) was used, with the following modifications: 5 *μ*g of DNA sample was fragmented by the NEBNext dsDNA Fragmentase (New England Biolabs, USA). Sequencing reaction was performed on the Illumina GAIIx platform with version 4 chemistry and version 4 flow cells according to the manufacturer's instructions. 76 base paired-end reads were generated. In order to remap the sequence reads to a reference human genome (UCSC NCBI37/hg19), we adopted the ELANDv2 software (Illumina Inc., USA). SNVs and indels detection were performed with run.pl script from CASAVA v1.6 (Illumina Inc., USA). To find variants from exon enrichment reads, the SNP Caller coverage cutoff option was turned off. SNVs and indels were compared with NCBI dbSNP v131 to distinguish known SNPs (those that had been deposited to dbSNP) and novel SNPs (those that were not in dbSNP). All the SNPs were annotated by comparing their position to other genomic features including gene regions.

### 2.3. GoldenGate Assay

Of 266,122 variants from NGS, 745 were expected to be functional (i.e., missense or nonsense). Of these functional variants, 467 were not found in a reference sequence in Genbank. Finally, of 467, we successfully designed 383 probes for GoldenGate Assay (Illumina Inc., USA) using the Illumina Assay Design Tool (Illumina design scores >0.4 and designable). Using this custom BeadChip, we searched disease-specific mutation among ten members of the present family with FAME including five affected, 85 unrelated individuals from the same community, which is an ancestral population of the proband, and 24 individuals from a diverse ethnic panel commercially provided by the Coriell Cell Repositories (Coriell Institute for Medical Research, Camden, NJ, USA). Genotype calls were made using the Genotyping module of the GenomeStudio software.

## 3. Results 

**Figure 2:**
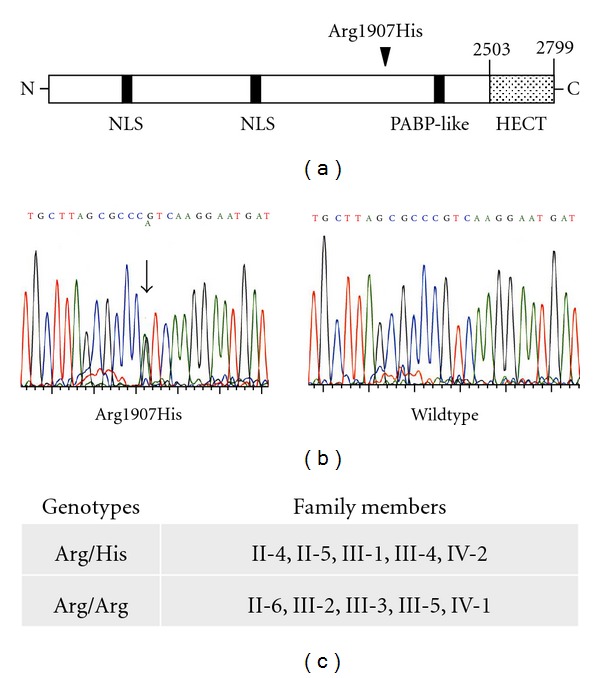
Arg1907His mutation in UBR5 in the family members affected with FAME. (a) A schematic representation of UBR5 structure, indicating the mutation site (arrowhead). HECT: homology to E6AP carboxy terminus, NLS: nuclear localization signal, PABP: poly(A)-binding proteins. (b) Electropherogram of the DNA sequence of the proband (left panel) showing a heterozygous G-to-A transition in exon 19 of the *UBR5 *gene (arrow), resulting in an Arg to His substitution at position 1907 in the protein. The right panel shows the wild type. (c) Genotypes of the mutation site in each family member. All the affected members have the Arg1907His mutation in UBR5, but the non-affected members do not. The symbols of the family members: see Figure 1.

**Figure 3:**
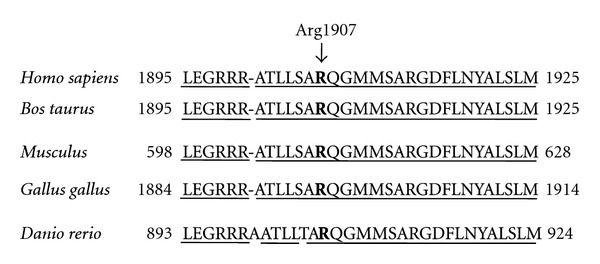
Arg-1907 (arrow) is an evolutionarily conserved aminoacid residue from *Homo sapiens *to *Danio rerio*. The identical aminoacid residues are underlined.

Genomic DNA from peripheral blood of the proband (III-1) was subjected to an exome analysis on NGS using the SureSelect Human All Exon System. All exons (37,354,942 bp) of the proband genome were sequenced and 36,768,760 bp (98.43%) were read. Among them, nucleotide variations were observed at 266,122 sites containing 467 functional variants (missense or nonsense). Of the 467 functional variants, the probes of 383 variants were successfully designed, and the GoldenGate Assay was performed for 10 family members, consisting of 5 patients with FAME (II-4, II-5, III-1, III-4, and IV-2) and 5 nonaffected members (II-6, III-2, III-3, III-5, and IV-1) (Figure 1). Of the 383 functional variants examined, only c.5720G>A mutation (p.Arg1907His) (NM_015902.5: exon 19) (chr8:103293724:GRCh37/hg19) in the *UBR5* gene (gene ID: 51366) was found in all of the affected individuals in the family, but not in any of the non-affected family members (Figure 2). The mutation was confirmed by conventional sequencing (Figure 2(b)). Such mutation was not found in any of the unrelated 85 healthy Japanese residents in the same community nor in any of the 24 individuals of various ethnicities. The Arg-1907 residue and its surrounding regions in UBR5 were highly conserved across species from *Homo sapiens *to *Danio rerio* (Figure 3).

## 4. Discussion 

In the present study, an exome analysis of the proband and the subsequent GoldenGate assay for the family members identified the c.5720G>A mutation (p.Arg1907His) in the *UBR5* gene only in the affected members in the FAME family, but not in the non-affected family members (Figure 2). No such mutation was found in any of the 109 persons examined, including the unrelated 85 healthy residents in the same community and the 24 subjects of various ethnicities. The conservation of the Arg-1907 residue in UBR5 across species suggests a functional importance of the residue (Figure 3). The *UBR5* gene was reported to be located in chromosome 8q22.3 [8], which is close to the reported locus linked to Japanese FAME families [2].

In European families with FAME, chromosome 2p11.1-q12.2 [3–6] or chromosome 5p15.31-p15 [7] has been mapped as the loci linked to FAME. Therefore, three, or more, genes are assumed to be responsible for FAME. This genetic heterogeneity in FAME is not surprising because in many genetic diseases including familial Alzheimer's disease and familial parkinsonism, a similar clinical phenotype can be produced by mutations in the different genes [9]. It seems likely that the proteins encoded by the causal genes for FAME may functionally be related with each other, and each may converge to the same biochemical pathway. The dysfunction of any of the FAME-associated proteins may cause a dysfunction of the pathway, resulting in a similar clinical manifestation.

*UBR5* is a human homolog of the *Drosophila melanogaster* tumor suppressor gene *hyperplastic discs* (*hyd*) [8, 10]. Human UBR5 is a huge protein with 2799 aminoacid residues (Figure 2(a)) [8]. The protein is localized mainly in the nucleus and has been shown to be ubiquitously expressed in various human tissues, including the brain [8]. UBR5 has the HECT (homology to E6AP carboxy terminus) domain at the C-terminus, which functions as E3 ubiquitin-protein ligase (Figure 2(a)) [8–11]. E3 ubiquitin-protein ligases are involved in protein degradation in the ubiquitin-proteasome system, which plays an important role in a variety of fundamental cell regulations, such as gene expression, stress response, DNA repair, and cell cycle [12]. Previous studies have shown that mutations in the genes encoding E3 ubiquitin-protein ligases can cause hereditary neurological diseases including Angelman syndrome [13] and autosomal recessive juvenile parkinsonism (ARJP) [14], both of which show tremor or tremulous involuntary movement as in FAME. 

In conclusion, we identified an FAME-associated mutation in the *UBR5* gene in the candidate region linked to Japanese FAME families. Further study is needed to clarify the significance of the mutant protein in the pathogenesis of FAME.

## Abbreviations

BAFME: Benign adult familial myoclonic epilepsy
FAME: Familial adult myoclonic epilepsy
UBR5: Ubiquitin protein ligase E3 component *n*-recognin 5.

## References

[1] Uyama E, Fu YH, Ptácek LJ (2005). Familial adult myoclonic epilepsy (FAME). *Advances in Neurology*.

[2] Mikami M, Yasuda T, Terao A (1999). Localization of a gene for benign adult familial myoclonic epilepsy to chromosome 8q23.3-q24.1. *American Journal of Human Genetics*.

[3] De Falco FA, Striano P, De Falco A (2003). Benign adult familial myoclonic epilepsy: genetic heterogeneity and allelism with ADCME. *Neurology*.

[4] Striano P, Chifari R, Striano S (2004). A new benign adult familial myoclonus epilepsy (BAFME) pedigree suggesting linkage to chromosome 2p11.1-q12.2. *Epilepsia*.

[5] Madia F, Striano P, Di Bonaventura C (2008). Benign adult familial myoclonic epilepsy (BAFME): evidence of an extended founder haplotype on chromosome 2p11.1-q12.2 in five Italian families. *Neurogenetics*.

[6] Saint-Martin C, Bouteiller D, Stevanin G (2008). Refinement of the 2p11.1-q12.2 locus responsible for cortical tremor associated with epilepsy and exclusion of candidate genes. *Neurogenetics*.

[7] Depienne C, Magnin E, Bouteiller D (2010). Familial cortical myoclonic tremor with epilepsy: the third locus (FCMTE3) maps to 5p. *Neurology*.

[8] Callaghan MJ, Russell AJ, Woollatt E, Sutherland GR, Sutherland RL, Watts CKW (1998). Identification of a human HECT family protein with homology to the *Drosophila* tumor suppressor gene hyperplastic discs. *Oncogene*.

[9] Lill CM, Bertram L (2011). Towards unveiling the genetics of neurodegenerative diseases. *Seminars in Neurology*.

[10] Mansfield E, Hersperger E, Biggs J, Shearn A (1994). Genetic and molecular analysis of hyperplastic discs, a gene whose product is required for regulation of cell proliferation in *Drosophila melanogaster* imaginal discs and germ cells. *Developmental Biology*.

[11] Honda Y, Tojo M, Matsuzaki K (2002). Cooperation of HECT-domain ubiquitin ligase hHYD and DNA topoisomerase II-binding protein for DNA damage response. *Journal of Biological Chemistry*.

[12] Jana NR (2012). Protein homeostasis and aging: role of ubiquitin protein ligase. *Neurochemistry International*.

[13] Matsuura T, Sutcliffe JS, Fang P (1997). De novo truncating mutations in E6-Ap ubiquitin-protein ligase gene (UBE3A) in Angelman syndrome. *Nature Genetics*.

[14] Shimura H, Hattori N, Kubo SI (2000). Familial Parkinson disease gene product, parkin, is a ubiquitin-protein ligase. *Nature Genetics*.

